# Dosimetric study of GZP6 ^60^Co high dose rate brachytherapy source

**DOI:** 10.1002/acm2.12362

**Published:** 2018-05-28

**Authors:** Qin Lei, Anjian Xu, Chengjun Gou, Yumei Wen, Donglin He, Junxiang Wu, Qing Hou, Zhangwen Wu

**Affiliations:** ^1^ Key Laboratory of Radiation Physics and Technology Ministry of Education Institute of Nuclear Science and Technology Sichuan University Chengdu China; ^2^ Nuclear Power Institute of China Chengdu Sichuan China; ^3^ Sichuan Cancer Hospital & Institute Chengdu Sichuan China

**Keywords:** ^60^Co, brachytherapy, dosimetry, Monte Carlo simulation

## Abstract

The purpose of this study was to obtain dosimetric parameters of GZP6 ^60^Co brachytherapy source number 3. The Geant4 MC code has been used to obtain the dose rate distribution following the American Association of Physicists in Medicine (AAPM) TG‐43U1 dosimetric formalism. In the simulation, the source was centered in a 50 cm radius water phantom. The cylindrical ring voxels were 0.1 mm thick for *r* ≤ 1 cm, 0.5 mm for 1 cm < *r* ≤ 5 cm, and 1 mm for *r* > 5 cm. The kerma‐dose approximation was performed for *r* > 0.75 cm to increase the simulation efficiency. Based on the numerical results, the dosimetric datasets were obtained. These results were compared with the available data of the similar ^60^Co high dose rate sources and the detailed dosimetric characterization was discussed.

## 1 INTRODUCTION

Monte Carlo (MC) simulation of electron and photon transport has been widely used in many areas of interest in medical physics, mainly in the development of the brachytherapy field. Calculation of kerma, absorbed dose, fluence, and related quantities at a geometric point is an important application of the Monte Carlo method. Many kinds of Monte Carlo simulation software have been successfully used for dosimetric studies, including Geant4, MCNP, EGSnrc (an extended and improved version of the EGS4), PENELOPE, and FLUKA.

The GZP6 ^60^Co afterloading high dose rate (HDR) unit (Nuclear Power Institute of China) is widely used at home and abroad. It is comprised of six different source designs with a stepping source (source number 6) and five nonstepping sources (source number 1–5). It mainly addresses intracavitary and interstitial applications and is considered an integral part of the treatment of cervical, vaginal, rectal, and esophageal cancers. In the literature, several investigations have been performed on the GZP6 ^60^Co HDR unit. In the study of Mesbahi et al.^1^, air kerma strengths of source numbers 1, 2, and 5 were obtained by in‐air measurements and a Farmer‐type ionization chamber. In a separate investigation, the radial dose functions of the three sources were calculated by Mesbahi et al.^2^ using the MC method and GZP6 TPS. Toossi et al.^3^ estimated the air kerma strength of GZP6 ^60^Co source number 3 by Monte Carlo simulation and in‐air measurements. The dose distribution for GZP6 ^60^Co stepping source was also calculated using the matrix shift method by Toossi et al.^4^ For the purpose of quality assurance, the dose distributions generated by GZP6 TPS were verified in another investigation.^5^

The dosimetric parameters of radioactive sources are crucial elements in clinical practice as they are important input data in treatment planning systems. Tabrizi et al. (2012) used the MCNP4C Monte Carlo code to obtain the dosimetric parameters of GZP6 ^60^Co sources. However, in the study of Vijande et al.^6^, it is indicated that the radial dose function from the study of Tabrizi et al. is inconsistent with other ^60^Co source data and is difficult to understand from a physical point of view. Hence, additional investigation of the dosimetric parameters of GZP6 ^60^Co sources is needed.

This work comprises a full and MC‐based dosimetry report for GZP6 ^60^Co source number 3 according to the recommendations of the American Association of Physicists in Medicine (AAPM) and the European Society for Radiotherapy and Oncology (ESTRO) on dose calculations for high‐energy (average energy higher than 50 keV) photon‐emitting brachytherapy sources.^7^, ^8^, ^9^ The calculated dosimetric parameters were compared with the available data of the similar ^60^Co high dose rate (HDR) sources^6^, ^10^, ^11^ and the detailed dosimetric characterization of GZP6 ^60^Co source number 3 was discussed.

## 2 MATERIAL AND METHODS

**Figure 1:**
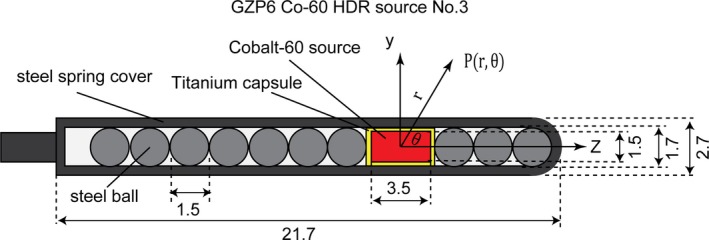
A schematic view of the GZP6 ^60^Co HDR source number 3. Dimensions are given in mm.

GZP6 ^60^Co brachytherapy source number 3 is composed of an active cylindrical ^60^Co pellet with a 3.5 mm length and a 1.5 mm diameter covered with a titanium layer with a thickness of 0.1 mm. The radioactive ^60^Co is uniformly distributed in the core. Several nonactive steel pellets 1.5 mm in diameter line up with the active cylindrical ^60^Co pellet. All the pellets described above were packaged in a steel spring cover with a thickness of 0.5 mm. The detailed information of this source is taken from published studies^2^, ^12^ and is illustrated in Fig. 1. The mass density and chemical composition of the materials are shown in Table 1.

**Table 1:** Mass density and composition of the materials of GZP6 ^60^Co HDR source number 3

Material: description	Mass density (g/cm^3^)	Composition (element/weight fraction)
Cobalt: source core	8.85	Co/1
Titanium: source capsule	4.54	Ti/1
Steel pellets: spacers in the source braid	7.9	Fe/0.71994, C/0.0005, Si/0.0072, Mn/0.0137, S/0.00011, P/0.00025, Cr/0.17, Ni/0.0822, Mo/0.0013, V/0.0006, Ti/0.0042
Steel: spring cover	6.999	Fe/0.7416, Ni/0.069, S/0.0001, Cr/0.167, C/0.0006, Mn/0.0062, Cu/0.0026, Al/0.0062, Mo/0.0015, Si/0.0052

The ^60^Co brachytherapy source emits two gamma rays with energies of 1.33 MeV and 1.17 MeV, mixed with β rays whose maximum energy and average energy is 0.318 MeV and 0.096 MeV, respectively.^8^ In the simulation, the β rays are neglected because of absorption in the source steel cover.^10^

In this study, the Monte Carlo code Geant4^13^ (Geant4.9.6.P02 development version) is used to simulate transport and interaction of gamma rays emitted from the GZP6 ^60^Co HDR brachytherapy source in water. The Evaluated Photon Data Library, 1997 Version (EPDL97) and Evaluated Electron Data Library (EEDL) cross‐section libraries were used for photons and electrons, respectively.^14^, ^15^ In the simulation, a spherical liquid water phantom with a 50‐cm radius was utilized to approximate an infinite water environment. The density used for the liquid water has been 0.998 g/cm^3^ as recommended in the TG‐43 U1 report.^7^ The GZP6 ^60^Co HDR source was accommodated in the center of the water phantom. Electronic equilibrium within 1% was reached for ^60^Co at distances greater than 7 mm from the source center.^16^ Thus, the kerma‐dose approximation was performed for *r* > 0.75 cm to speed up calculations.^17^ Dose and collisional kerma rate distributions were used to derive the final dosimetric parameters as described in the AAPM TG‐43U1 report.^7^

The dose distribution of the source was calculated within the radial distance of 20 cm. The cylindrical ring voxels were 0.1 mm thick for *r* ≤ 1 cm, 0.5 mm for 1 cm < *r* ≤ 5 cm, and 1 mm for *r* > 5 cm, which can provide high‐resolution dosimetry. The cutoff energy was set to 10 keV for both photons and electrons. Collisional kerma and absorbed dose were obtained in cylindrical (*y*,* z*) and spherical (*r*, θ) coordinates. The coordinate axes used are shown in Fig. 1. The air kerma strength was calculated in a separate simulation with the source surrounded by vacuum, except for a cylindrical air cell of 0.1 cm in diameter and 0.1 cm in height at *r* = 10 cm. As clarified in the TG‐43U1S1 report,^18^ dry air (0% humidity) is recommended for air kerma strength in contrast to the TG‐43U1 report which recommended air at 40% relative humidity. 6 × 10^9^ photon histories (*r* ≤ 0.75 cm) were simulated to score dose. 10^9^ photon histories (*r* > 0.75 cm) were simulated to score kerma.

## 3 RESULTS AND DISCUSSION

The air kerma strength calculated for GZP6 ^60^Co source number 3 is 3.004 × 10^−7^ cGy cm^2^ h^−1^ Bq^−1^ with a statistical uncertainty of 0.14%. The dose rate constant of GZP6 ^60^Co source number 3 obtained is 1.088 ± 0.002 cGy h^−1^ U^−1^ (with *k* = 1), which is comparable to the available data of the similar ^60^Co HDR sources (see Table 2). The radial dose function values and the anisotropy function values of the GZP6 ^60^Co source number 3 are provided in Table 3 and Table 4, respectively. In Fig. 3, the anisotropy function results of GZP6 ^60^Co source number 3 are plotted vs polar angle at the selected radial distances. In addition, the along‐away data are shown in Table 5.

**Table 2:** Comparison of dose rate constant values for the comparable ^60^Co HDR sources

Source type	Λ (cGy h^−1^ U^−1^)	Λ/G (*r*_0_, θ_0_)
The new BEBIG ^60^Co^10^	1.087 ± 0.0011	1.098 ± 0.0011
Ralston Type‐2 ^60^Co^11^	1.101 ± 0.005	1.105 ± 0.005
Flexisource ^60^Co^6^	1.085 ± 0.003	1.096 ± 0.003
GZP6 ^60^Co source num. 3 (this work)	1.088 ± 0.002	1.099 ± 0.002

**Table 3:** Radial dose function calculated for the GZP6 ^60^Co HDR source number 3

*r* (cm)	*g*_L_ (r)
0.25	1.05
0.33	1.035
0.5	1.022
0.75	1.008
1	1
1.5	0.991
2	0.984
3	0.969
4	0.953
5	0.937
6	0.921
7	0.904
8	0.887
9	0.869
10	0.853
12	0.816
15	0.759
20	0.664

**Table 4:** Anisotropy function calculated for the GZP6 ^60^Co HDR source number 3

θ(°)	*r* (cm)																	
	0.25	0.33	0.5	0.75	1	1.5	2	3	4	5	6	7	8	9	10	12	15	20
0	–	–	–	0.874	0.874	0.876	0.885	0.885	0.887	0.888	0.894	0.896	0.901	0.903	0.907	0.915	0.927	0.924
1	–	–	–	0.873	0.871	0.874	0.883	0.885	0.887	0.891	0.895	0.897	0.901	0.904	0.906	0.913	0.920	0.925
2	–	–	–	0.872	0.869	0.873	0.881	0.886	0.888	0.893	0.896	0.898	0.901	0.905	0.904	0.911	0.916	0.926
3	–	–	–	0.877	0.880	0.883	0.888	0.891	0.892	0.895	0.897	0.900	0.903	0.906	0.905	0.911	0.916	0.919
4	–	–	–	0.881	0.883	0.889	0.892	0.895	0.897	0.900	0.904	0.906	0.908	0.911	0.910	0.913	0.915	0.920
5	–	–	–	0.884	0.891	0.893	0.895	0.897	0.898	0.899	0.900	0.902	0.906	0.907	0.906	0.912	0.916	0.921
6	–	–	–	0.900	0.903	0.901	0.897	0.896	0.898	0.901	0.904	0.906	0.908	0.910	0.910	0.914	0.918	0.924
7	–	–	–	0.909	0.906	0.903	0.900	0.899	0.901	0.903	0.906	0.907	0.910	0.912	0.912	0.915	0.919	0.924
8	–	–	–	0.921	0.909	0.904	0.902	0.902	0.904	0.906	0.908	0.909	0.912	0.914	0.914	0.918	0.920	0.925
9	–	–	–	0.925	0.910	0.905	0.904	0.905	0.906	0.909	0.910	0.912	0.915	0.916	0.917	0.921	0.924	0.928
10	–	–	–	0.931	0.911	0.906	0.907	0.908	0.909	0.912	0.912	0.916	0.918	0.919	0.920	0.925	0.929	0.931
15	–	–	–	0.934	0.930	0.930	0.930	0.931	0.931	0.932	0.933	0.934	0.936	0.938	0.938	0.939	0.941	0.943
20	–	–	0.963	0.943	0.952	0.952	0.951	0.951	0.951	0.952	0.952	0.952	0.953	0.955	0.954	0.955	0.959	0.963
25	–	–	0.962	0.964	0.967	0.966	0.965	0.966	0.966	0.966	0.966	0.966	0.967	0.967	0.966	0.967	0.967	0.970
30	–	–	0.972	0.973	0.976	0.977	0.975	0.976	0.975	0.975	0.975	0.975	0.976	0.977	0.975	0.977	0.976	0.976
40	0.991	0.986	0.983	0.989	0.987	0.988	0.987	0.987	0.987	0.987	0.987	0.986	0.986	0.987	0.985	0.986	0.984	0.984
50	1.000	0.995	0.993	0.990	0.994	0.995	0.994	0.994	0.995	0.994	0.994	0.994	0.994	0.995	0.993	0.994	0.992	0.991
60	1.000	0.997	0.995	0.989	0.997	0.997	0.996	0.997	0.996	0.996	0.995	0.995	0.996	0.996	0.993	0.994	0.997	0.993
70	1.000	0.998	0.998	0.983	1.000	0.999	0.998	0.999	0.999	0.998	0.998	0.997	0.998	0.999	0.998	0.997	0.997	0.997
80	1.000	1.000	0.998	1.000	1.000	1.000	1.000	1.000	0.999	0.998	0.999	0.998	0.998	0.999	0.998	0.997	1.000	1.000
90	1	1	1	1	1	1	1	1	1	1	1	1	1	1	1	1	1	1
100	1.000	1.000	0.999	1.000	1.000	1.000	1.000	1.000	0.999	0.999	0.999	0.998	0.998	0.999	0.998	0.999	1.000	1.000
110	1.000	1.000	0.998	0.986	0.999	0.998	0.999	0.999	0.999	0.998	0.999	0.998	0.998	0.998	0.998	0.997	0.998	0.997
120	0.998	0.996	0.995	0.991	0.997	0.997	0.996	0.997	0.997	0.997	0.996	0.996	0.996	0.995	0.994	0.995	0.997	0.995
130	1.000	0.996	0.994	0.991	0.995	0.996	0.995	0.995	0.994	0.995	0.995	0.995	0.995	0.995	0.994	0.995	0.993	0.994
140	0.996	0.988	0.984	0.985	0.987	0.988	0.988	0.989	0.988	0.988	0.988	0.986	0.987	0.989	0.987	0.988	0.986	0.986
150	–	–	0.971	0.974	0.977	0.977	0.977	0.976	0.976	0.976	0.976	0.976	0.976	0.978	0.978	0.978	0.978	0.979
155	–	–	0.961	0.964	0.967	0.968	0.968	0.967	0.967	0.968	0.968	0.968	0.969	0.970	0.970	0.970	0.971	0.973
160	–	–	0.946	0.952	0.954	0.954	0.954	0.953	0.954	0.955	0.956	0.956	0.957	0.960	0.960	0.961	0.965	0.969
165	–	–	–	–	0.933	0.933	0.932	0.931	0.929	0.930	0.931	0.931	0.933	0.934	0.934	0.937	0.941	0.944
170	–	–	–	–	–	0.883	0.885	0.884	0.885	0.887	0.887	0.890	0.894	0.895	0.896	0.901	0.905	0.907
171	–	–	–	–	–	0.863	0.863	0.863	0.864	0.868	0.870	0.872	0.875	0.876	0.878	0.882	0.886	0.890
172	–	–	–	–	–	0.851	0.851	0.851	0.853	0.857	0.860	0.862	0.866	0.869	0.869	0.873	0.875	0.880
173	–	–	–	–	–	0.820	0.820	0.821	0.824	0.829	0.834	0.835	0.838	0.840	0.841	0.847	0.853	0.863
174	–	–	–	–	–	0.797	0.797	0.799	0.804	0.809	0.815	0.820	0.824	0.829	0.832	0.842	0.848	0.856
175	–	–	–	–	–	–	0.776	0.778	0.785	0.792	0.799	0.804	0.812	0.817	0.818	0.828	0.838	0.849
176	–	–	–	–	–	–	0.774	0.774	0.780	0.787	0.794	0.800	0.806	0.812	0.815	0.824	0.833	0.844
177	–	–	–	–	–	–	0.770	0.772	0.779	0.786	0.794	0.794	0.803	0.808	0.809	0.819	0.830	0.841
178	–	–	–	–	–	–	0.769	0.770	0.778	0.784	0.793	0.793	0.802	0.806	0.807	0.817	0.829	0.839
179	–	–	–	–	–	–	0.766	0.768	0.776	0.781	0.791	0.792	0.801	0.805	0.807	0.818	0.828	0.835
180	–	–	–	–	–	–	0.770	0.770	0.777	0.779	0.792	0.793	0.802	0.805	0.810	0.820	0.830	0.840

**Table 5:** Dose rate results (cGy h^−1^ U^−1^) around the GZP6 ^60^Co HDR source number 3

*z* (cm)	*y* (cm)												
	0	0.25	0.33	0.5	0.75	1	1.5	2	3	4	5	6	7
−7	0.016	0.016	0.016	0.016	0.016	0.017	0.018	0.018	0.016	0.015	0.013	0.011	0.009
−6	0.022	0.023	0.022	0.022	0.023	0.024	0.024	0.024	0.022	0.019	0.016	0.013	0.011
−5	0.032	0.033	0.032	0.033	0.035	0.036	0.035	0.034	0.029	0.024	0.020	0.016	0.013
−4	0.051	0.051	0.051	0.054	0.056	0.057	0.055	0.050	0.041	0.032	0.024	0.019	0.015
−3	0.091	0.091	0.094	0.101	0.103	0.101	0.092	0.080	0.057	0.041	0.030	0.022	0.017
−2	0.207	0.223	0.232	0.237	0.228	0.210	0.170	0.132	0.081	0.052	0.035	0.025	0.019
−1.5	–	0.419	0.428	0.417	0.379	0.330	0.238	0.171	0.094	0.057	0.038	0.026	0.019
−1	–	0.986	0.962	0.868	0.697	0.544	0.333	0.215	0.106	0.061	0.039	0.027	0.020
−0.5	–	3.688	3.176	2.234	1.355	0.875	0.434	0.254	0.115	0.064	0.041	0.028	0.020
0	–	16.088	9.609	4.323	1.935	1.088	0.482	0.270	0.118	0.065	0.041	0.028	0.020
0.5	–	3.688	3.176	2.234	1.355	0.874	0.434	0.254	0.115	0.064	0.041	0.028	0.020
1	0.988	0.983	0.959	0.867	0.696	0.544	0.333	0.215	0.106	0.061	0.039	0.027	0.019
1.5	0.429	0.432	0.430	0.416	0.378	0.329	0.238	0.171	0.094	0.057	0.038	0.026	0.019
2	0.238	0.241	0.240	0.237	0.227	0.210	0.169	0.132	0.081	0.052	0.035	0.025	0.018
3	0.105	0.105	0.105	0.105	0.104	0.101	0.091	0.080	0.057	0.041	0.030	0.022	0.017
4	0.057	0.059	0.058	0.058	0.058	0.057	0.054	0.050	0.040	0.032	0.024	0.019	0.015
5	0.036	0.037	0.037	0.037	0.036	0.036	0.035	0.034	0.029	0.024	0.020	0.016	0.013
6	0.025	0.025	0.025	0.025	0.025	0.025	0.024	0.024	0.022	0.019	0.016	0.013	0.011
7	0.018	0.018	0.018	0.018	0.018	0.018	0.018	0.018	0.016	0.015	0.013	0.011	0.009

**Figure 2:**
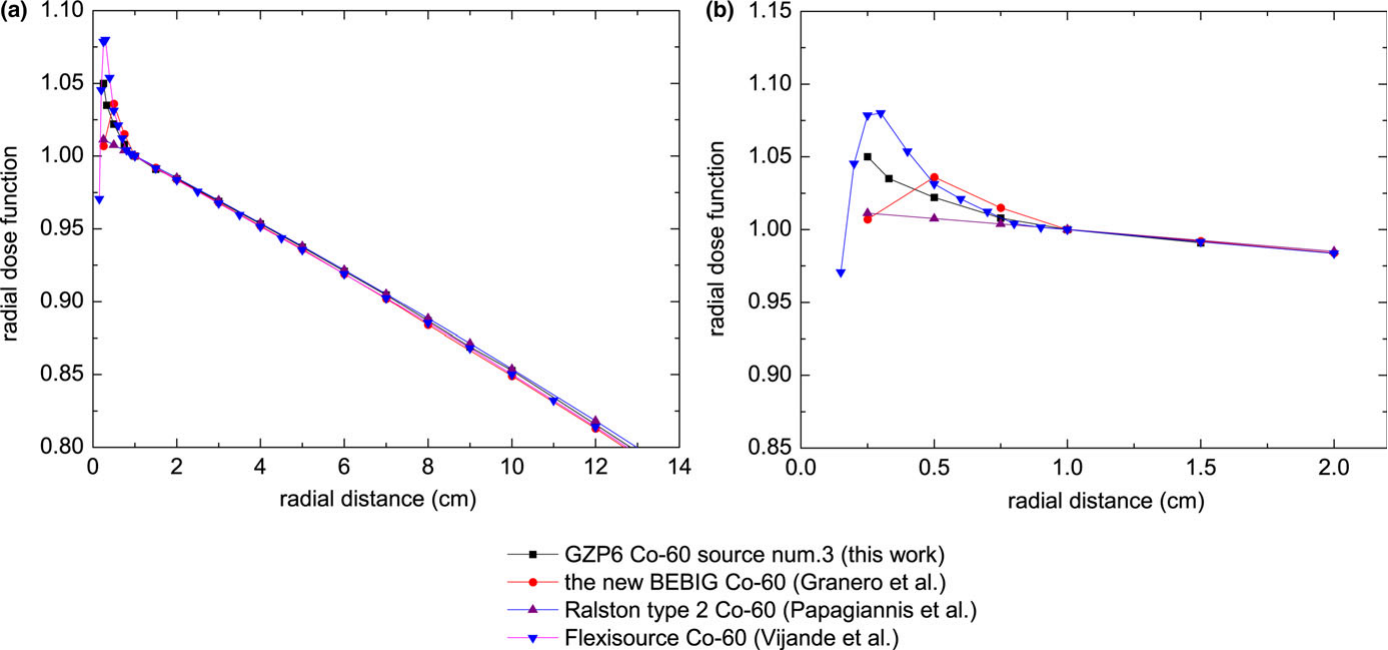
(a) Radial dose function of ^60^Co source models. (b) Zoom‐in at short distances from the source.

The radial dose function values of the GZP6 ^60^Co source number 3 were compared with corresponding data from the relevant literature (see Fig. 2). It is observed that the curves of the radial dose functions of the source models match well for *r* > 1 cm and small differences exist for *r* < 1 cm. These differences are caused by varying degrees of photon absorption and scattering in the sources.^8^ In general, the radial dose functions do not depend significantly on source dimensions and encapsulation designs.^11^

**Figure 3:**
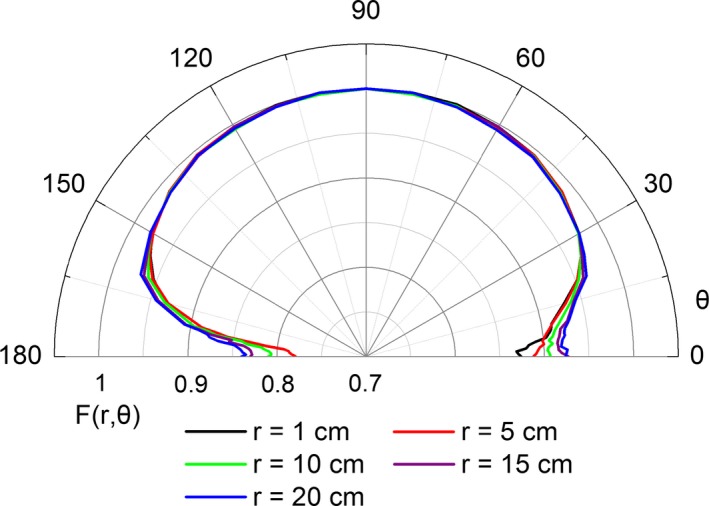
Anisotropy function of the GZP6 ^60^Co HDR source number 3 at selected radial distances.

As shown in Table 4 and Fig. 3, the anisotropy function values of GZP6 ^60^Co source number 3 are nearly uniform for polar angles 30° ≤ θ ≤ 90°. For example, the anisotropy function values are around 0.998 for θ = 70° and around 0.994 for θ = 50°. However, a strong dependence on radial distance was observed for θ < 30°. As described in Ref. 8, the anisotropy function values decrease for polar angles close to the long axis (see Fig. 3), which is caused by the oblique filtration within the source structure.

According to AAPM TG‐43U1 recommendation,^7^ the uncertainty of the final dose rate values has been estimated, including statistical (A) and systemic uncertainty (B). The statistical uncertainty in water phantom calculations is less than 0.6% for all the points, except at the points located near the longitudinal axis, where it is less than 1.1%. For the simulation of the air kerma strength, the statistical uncertainty is 0.14%. This gives us a combined A‐type uncertainty of 0.6% for all the points, except near longitudinal axis points where the combined A‐type uncertainty is about 1.1%. The type B uncertainty is negligible for ^60^Co sources.^10^, ^19^ Thus, the total uncertainty is 0.6% for all the points except for the longitudinal axis points, which is 1.1%.

## 4 CONCLUSIONS

In this study, the Geant4 MC code was used to study the dose rate distribution around the GZP6 ^60^Co HDR source number 3. The dosimetric parameters for this source are obtained as required by AAPM and ESTRO. The calculated dose rate constant of the GZP6 ^60^Co source number 3 is 1.088 ± 0.002 cGy h^−1^ U^−1^ and the radial dose functions are consistent with the available data of the similar ^60^Co HDR sources. In addition, a 2D rectangular dose rate table is presented.

## CONFLICTS OF INTEREST

None declared.
